# Direct Contact Membrane Distillation of Dairy Process Streams

**DOI:** 10.3390/membranes1010048

**Published:** 2011-01-04

**Authors:** Angela Hausmann, Peter Sanciolo, Todor Vasiljevic, Elankovan Ponnampalam, Nohemi Quispe-Chavez, Mike Weeks, Mikel Duke

**Affiliations:** 1 ISI-Institute for Sustainability and Innovation, Victoria University, Melbourne, Victoria, 3030, Australia; E-Mails: peter.sanciolo@vu.edu.au (P.S.); todor.vasiljevic@vu.edu.au (T.V.); mikel.duke@vu.edu.au (M.D.); 2 Dairy Innovation Australia Ltd., Werribee, Victoria, 3030, Australia; E-Mails: eponnampalam@dairyinnovation.com.au (E.P.); nquispe-chavez@dairyinnovation.com.au (N.Q.C.); mweeks@dairyinnovation.com.au (M.W.)

**Keywords:** direct contact membrane distillation, milk dairy

## Abstract

Membrane distillation (MD) was applied for the concentration of a range of dairy streams, such as whole milk, skim milk and whey. MD of a pure lactose solution was also investigated. Direct contact MD (DCMD) mode experiments were carried out in continuous concentration mode, keeping the warm feed/retentate and cold permeate stream temperatures at 54 °C and 5 °C respectively. Performance in terms of flux and retention was assessed. The flux was found to decrease with an increase of dry-matter concentration in the feed. Retention of dissolved solids was found to be close to 100% and independent of the dry-matter concentration in the feed. Fourier Transform Infrared Spectroscopy (FTIR) of the fouled membranes confirms organics being present in the fouling layer.

## 1. Introduction

Membrane technologies are widely applied to dairy processing, but they rely on the generation of large pressure differences across the membrane [1]. This results in a high amount of electrical energy being consumed for high pressure pumps. Direct contact membrane distillation (DCMD) is a thermal membrane process performing at low grade feed temperatures allowing the utilization of waste heat from industrial systems and even solar energy [2].

Membrane distillation (MD) is not yet widely applied on industrial scale but the number of investigations on practical applications is ever increasing. These can be differentiated into the following two categories:

(1) Water recovery or production of purified water.
(2) Concentration of aqueous streams at high retention rates.

The combination of both aspects is highly desirable to make full use of the advantages MD bears.

Membrane distillation is currently utilized for production of demineralized water [3-5]. Its use for fruit juice [6-8], sugar syrup [9] and ginseng extract [10] concentration has been proposed and investigated, but in the field of dairy processing few papers have been published on the concentration of whey proteins [11]. In 1987, Chlubek *et al.* [12] reported the use of MD for concentrating milk whey. They pre-treated whey by precipitating proteins, to obtain a protein free solution. More recently, Christensen *et al.* [11] used the direct contact MD method on a polypropylene membrane tube to increase dry-matter of a whey protein concentrate up to 34% total solids. The motivation of their tests was to improve product quality by using milder temperatures compared to standard evaporation (55 °C versus 70 °C) which reduces partial denaturation of whey proteins. They conclude that DCMD is a possibility for industrial application but higher fluxes need to be achieved. In the present study different module geometry has been tested, using flat-sheet membranes for concentration by DCMD.

## 2. Theory

**Figure 1:**
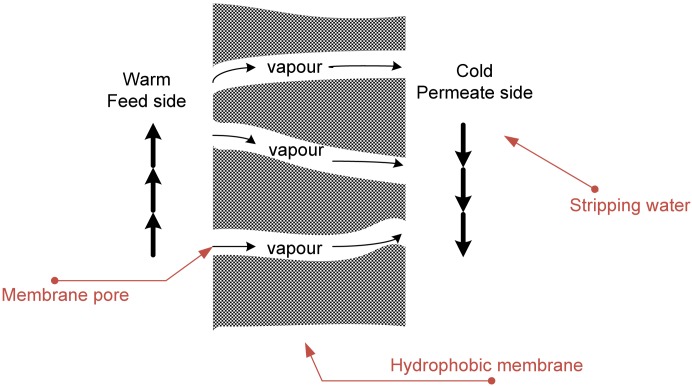
Schematic of DCMD, adapted from [14].

Membrane distillation is an evaporation/condensation process of a volatile solvent through a hydrophobic porous membrane driven by the partial vapour pressure difference across the membrane [13]. The overall concept of direct contact membrane distillation (DCMD) is illustrated in Figure 1. In a DCMD process, the temperature difference and corresponding vapour pressure difference across the membrane is created by the direct contacting of a liquid cooler than the feed on the permeate side of the membrane. Vapours diffuse through the pores to the cooler surface where they condense.

The membrane material needs to be hydrophobic, so that only water in the vapour phase can enter and pass the pores. As a result, solute separation is very high for aqueous solutions of non-volatile compounds [15]. Membrane materials commonly employed for MD are polypropylene (PP) or polytetrafluoroethylene (PTFE). Performance of this process is essentially unaffected by osmotic pressure at high concentrations and can theoretically reach higher concentrations than pressure driven membrane processes. Nevertheless, temperature polarization lowers performance of this process with increasing concentration and a new issue arises from membrane wettability as a result from feed components entering membrane pores in a process where only vapour is supposed to pass the membrane. In the case of emulsions, a hydrophobic membrane may get wetted by oil present in the feed solution. Gryta *et al.* [16] even proposed the application of membrane distillation for the concentration of oil-water emulsions as in membrane distillation both the oil phase and water phase of emulsions cannot flow through the pores filled with air of the hydrophobic membrane. As long as the membrane pores are smaller than the oil droplets in the feed solution and the oil droplets do not break causing the formation of a phase with free oil, the separation of emulsions by MD is possible. Work like this clearly indicates that the virtues of this relatively new process to dairy industry can only be found by realistic experimental trials.

**Figure 2:**
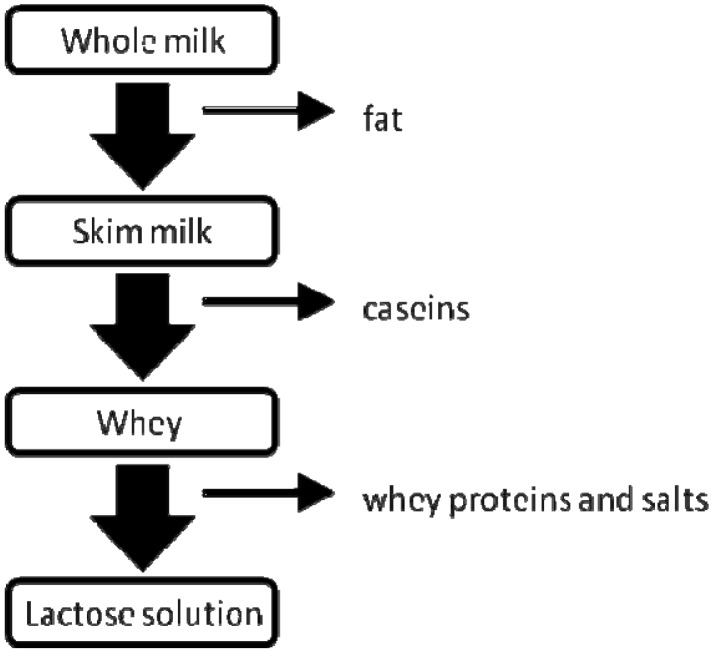
Simplified relation of dairy streams tested for DCMD to estimate influence of different milk components on overall process performance.

The interactions of dairy ingredients with membrane materials have been broadly studied for membrane materials used in filtration processes, MD however requires the use of hydrophobic membranes and the interactions of dairy ingredients with these polymeric membrane materials have not yet been investigated. Figure 2 shows the feed streams used in the current study and their major compositional differences.

In this study, performance results of direct contact membrane distillation for whole milk, skim milk, whey and a lactose powder solution were compared. The aim of this work was to test the possibility of MD application in dairy processing, with the main focus on gaining knowledge about the influence of the main dairy components in this new technology to help finding its best applications for water recovery within dairy processes.

## 3. Experimental Section

### 3.1. Lab-scale Direct Contact Membrane Distillation Set-up

**Figure 3:**
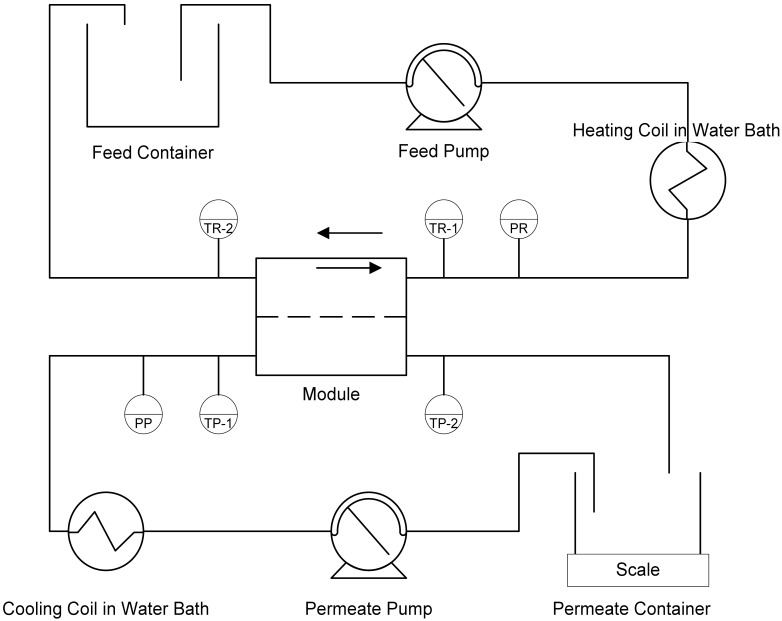
Laboratory scale DCMD set-up.

The experimental set-up scheme is presented in Figure 3. Polytetrafluorethylene (PTFE) flat-sheet membranes of 0.5 μm nominal pore-size were used in a laboratory scale Osmonics SEPA CF module housing using an effective membrane area of 0.014 m^2^. A peristaltic pump with two heads was used to provide a steady flow equivalent to a linear velocity of 0.047 m·s^−1^ on both sides of the membrane. The inlet pressure and all four inlet and outlet temperatures, as well as the permeate weight were recorded continuously. The flux was calculated from the permeate weight gain. The feed or retentate temperature at the module-inlet was kept at 54 °C and the permeate/stripping water temperature was maintained at 5 °C. The high temperature difference was chosen to investigate maximum performance of MD and reach high dry matter contents, while still remaining below the temperature of potential protein denaturation.

### 3.2. Experimental Procedure

Direct contact membrane distillation experiments were set up in a continuous concentration batch mode. The feed and distillate streams flowed in the module in counter-current mode, each circulating in a closed loop. The conductivity of the permeate was monitored throughout the experiment to register changes in retention or leaking of the membrane. Permanent recording of the feed pH was necessary to ensure that a drop in pH was not the cause of a drop in flux, and that this drop in pH did not alter fouling layer composition. Experiments were stopped when flux had dropped to zero or when the pH had dropped due to bacterial growth. For every experiment a new membrane was used.

### 3.3. Tested Solutions

The feed solutions tested were whey and skim milk powders that had been dissolved in deionised water, 5.5% (w/v) for the whey powder solution and 9.5% (w/v) for the skim milk one. More concentrated solutions were prepared to follow on from the end concentration of previous experiments. Fresh whole milk was also tested.

### 3.4. Dry-Matter Analysis by Sea-Sand Method

Feed and retentate samples were homogenized with sea-sand and dried at 110 °C in an oven for at least 14 hours. The total solids content was calculated by the absolute difference in weight before and after drying following the Standard Methods for the Examination of Dairy Product [17].

### 3.5. Total Organic Carbon Analysis

Permeate and retentate samples were analyzed for total organic carbon using a Total Organic Carbon Analyzer (Shimadzu V_CSH_).

### 3.6. FTIR of Fouled Membranes

The absorbance of functional groups on the surface layer of fouled and a clean membrane were measured using a Fourier Transform-IR spectrophotometer (Shimadzu IRAffinity 1), fitted with a specular reflectance accessory attachment. Clean and fouled membrane samples were rinsed with MQ water and air-dried before analysis. Sixty-four scans were completed, measuring absorbance across the 400–2000 cm^−1^ wavenumber range, with a sensitivity of 4.0 cm^−1^.

## 4. Results & Discussion

### 4.1. Water Permeation and MD Performance

**Figure 4:**
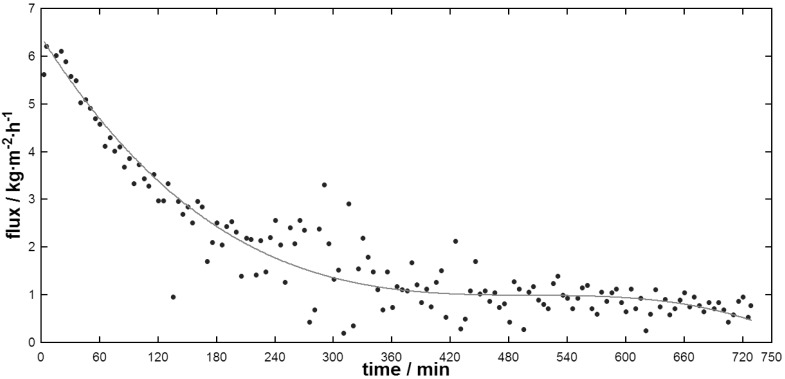
Flux during whole milk concentration by DCMD.

As shown in Figure 4, during the membrane distillation of whole milk, the flux decreased continuously in the first 5 to 6 hours, then remained stable at around ∼1 kg·m^−2^·h^−1^. The total solids content increased from 14.74% to 18.04%.

After the experiment the membrane appeared slightly wetted which was most likely due to the higher fat content of whole milk interacting with the hydrophobic PTFE surface. Evaporation of water through the membrane would in turn be inhibited as the membrane surface became covered in fats. This observation was similar to that of Gryta *et al.* [16] who reported that membrane wetting by fats in the feed solution was more likely with bigger and therefore unstable fat globules. Unhomogenized milk contains such unstable fat globules and 90% of the milk lipids are in globules with a diameter of 1 to 15 μm [18]. As shown in Figure 4, flux reduction was more severe at the beginning of the experiment, which could be due to fouling establishment. Also possible is that the bigger and more unstable fat globules break and wet the membrane until remaining fat globule size and stability is sufficient for the slight increase in total solids. Homogenization might be necessary to reduce wetting and improve flux as the resulting secondary milk fat globule membrane mainly consisting of proteins and the smaller globule sizes might increase globule stability. However, even with more stable fat globules, the likelihood of penetration of fat into the membrane pores increases with increasing fat concentration during MD, which limits applicability of MD for fatty streams [16].

**Figure 5:**
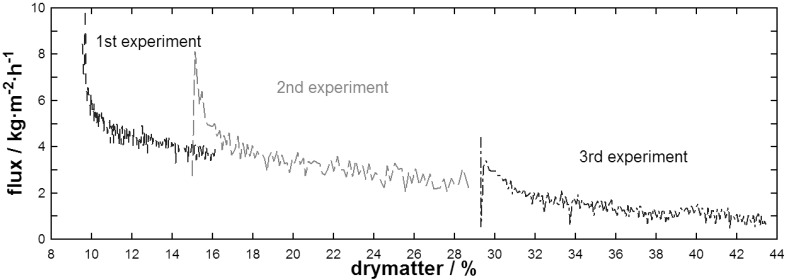
Flux during DCMD of skim milk powder solutions at different initial concentrations.

MD flux depending on concentration of skim milk powder solutions at different initial concentrations of the feed is presented in Figure 5. Based on the continuity of flux between the end of one experiment to the start of the next, it was obvious that the overall reduction of flux occurred as a result of dry-matter concentration of the feed solution. This resulted from an increasing temperature polarization. The effect of polarization at the membrane surface strongly depends on hydrodynamic conditions applied, as well as the viscosity of the feed solution. Hydrodynamic conditions have been constant for all experiments and a final dry-matter concentration of 43.5% has been achieved with set parameters. Changes in viscosity of the feed solution are reflected in the module inlet pressure which increased from 4 to 30 kPa over the entire range of all three experiments.

Flux at the beginning of experiments dropped exponentially before entering the same linear slope for all three experiments. This initial rapid decline in flux was clearly a fouling establishment associated with using fresh membranes, and can be explained by the binding of feed components to the hydrophobic membrane. This strongly bound fouling layer remained constant after a first establishment phase so that flux then dropped linearly as a result of increasing concentration. One experiment with real skim milk, provided from a local dairy plant, and one with reconstituted skim milk powder solution starting at 20% dry-matter were also performed (data not shown), both showed similar flux over dry-matter behavior as presented in Figure 5.

**Figure 6:**
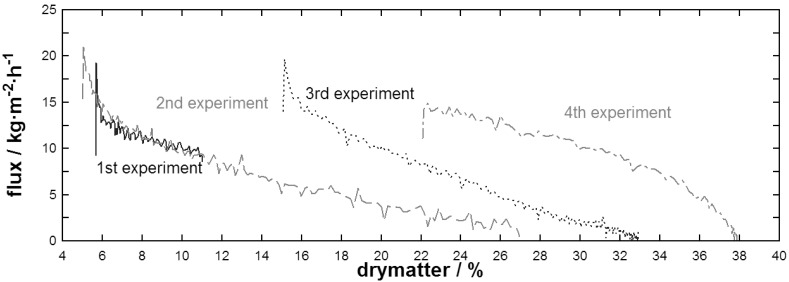
Flux during DCMD of a whey powder solution at different initial concentrations.

Figure 6 shows the flux behavior of whey powder solutions during DCMD. Here, the rate of flux decline was found to depend strongly on the initial dry-matter concentration. This indicated that flux not only declined as a matter of increasing concentration and resulting temperature polarization as found in Figure 5, but also as a result of time dependant fouling. Except for the initial establishment phase, flux over dry-matter dropped more or less linearly for the first 3 experiments. Mechanisms responsible for a flux decline over time might be due to the hydrophobic/hydrophilic character of the whey protein surface and conformational changes starting at temperatures below 60 °C over a long processing time as in the present case [19]. Also, feed inlet pressure did not increase linearly with dry-matter concentration but reached 30 kPa at the end of both, the 2nd and 3rd experiments indicating a viscosity increase that reduced turbulence at the membrane surface. After a first linear phase, flux of the 4th experiment decreased rapidly to zero, possibly due to exceeding lactose solubility limits. This is also indicated by the increase in feed inlet pressure up to 80 kPa and the observation of crystals on the membrane surface after the membrane has been dismounted.

At a dry-matter concentration of 10%, flux of the whey powder solution was around 10 kg·m^−2^·h^−1^ which is twice the flux of skim milk (around ∼5 kg·m^−2^·h^−1^) indicating that caseins reduce performance of DCMD with the given membrane material. However, flux behavior of the skim milk powder solution did not seem to be subjected to time as performance reduction was linear with increasing dry-matter concentration over a range of experiments.

**Figure 7:**
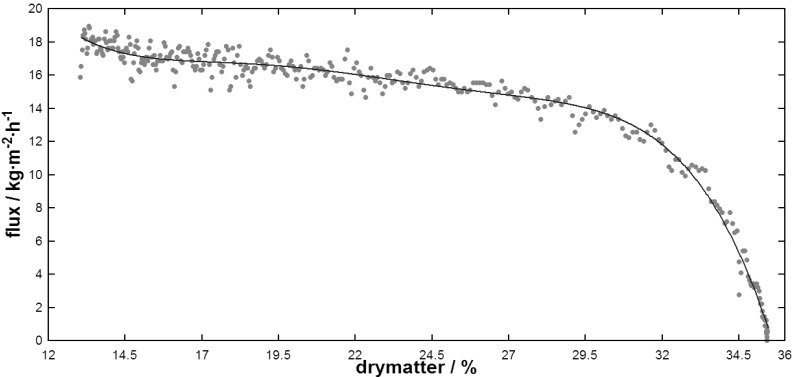
Flux during DCMD of a lactose powder solution.

As Figure 7 shows, lactose could be concentrated up to 35% dry-matter under given hydrodynamic conditions. Flux reduction was almost linear up to 30% total solids then falling exponentially along with a substantial increase of feed inlet pressure to 90 kPa. Limited solubility of lactose at the cooler membrane surface compared to the bulk feed stream could have contributed to this reduction as observed by the formation of crystals on the membrane surface after dismounting the membrane from the rig. This again was a consequence of temperature polarization as a 30% lactose solution at 54 °C is still in the solubility range of lactose [20].

As shown in Table 1, retention of total organic carbon was found to be between 99.2% and 99.9% for all feed solutions, showing the potential to produce a high quality permeate even when reaching high concentrations on the retentate side. This also confirmed that despite a slight membrane wetting during concentration of whole milk, milk fat did not pass the membrane.

**Table 1:** TOC-retention at end of experiments and permeate conductivity at beginning and end of experiments.

**Experiment**	**TOC retention**	**Permeate conductivity/μS**
**Whole milk–**experiment	99.97%	6.8–50.6
**Skim milk**–experiment 1	99.98%	2.1–7.9
**Skim milk**–experiment 2	99.99%	8.0–10.7
**Skim milk**–experiment 3	99.98%	1.6–15.2
**Whey**–experiment 1	99.98%	8.2–92.8
**Whey**–experiment 2	99.97%	5.6–6.8
**Whey**–experiment 3	99.95%	7.7–16.7
**Whey**–experiment 4	99.67%	6.2–185.5
**Lactose–**experiment	99.24%	2.1–30.3

### 4.2. FTIR

**Figure 8:**
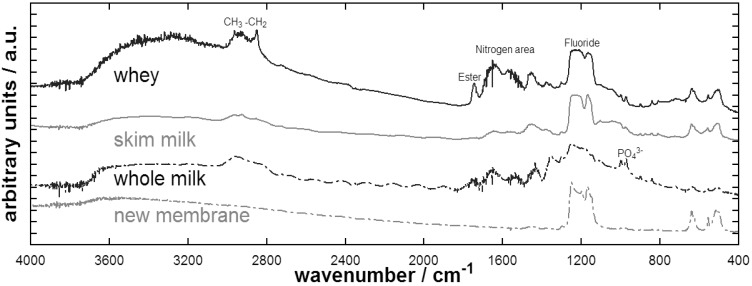
FTIR of fouled membranes and a new membrane.

As shown in Figure 8, the FTIR showed peaks at wave numbers corresponding to carbohydrates and in the region of nitrogen. These functional groups indicate the presence of proteins and organics in general in the remaining fouling layer. Such peaks were stronger for the whey fouled membrane, most probably reflecting its higher extent of fouling. Also, one peak corresponding to ester groups was more distinguished for the whey fouled membrane, which might be related to conformational changes of whey proteins.

The membrane polymer is represented in the peaks corresponding to fluoride. Here, the new membrane showed sharp peaks around 1,200 cm^−1^, whereas the fouled membranes peaks overlapped, presumably due to the fouling layer. FTIR of the whole milk membrane showed interferences, probably due to the extent of fouling. Here the peak corresponding to wavelengths of fluoride are overlapped by other peaks, indicating that the fouling layer covered the membrane surface. This membrane also showed peaks in the area corresponding to phosphate revealing that inorganic fouling also occurred.

The presented results allow an initial estimation of possible MD applications within the dairy industry. A very good separation of a series of solutions is indicated. However, results of the present work show that fouling issues need to be overcome (or appropriate cleaning strategies designed) before a commercial implementation of this process is viable on such streams. DCMD performance decreased with increasing dry-matter concentration. However, flux and achievable dry-matter contents can be increased by improving hydrodynamic factors such as feed and permeate flow rate. Still, concentrations achieved for the skim milk and whey powder solutions under the applied conditions on the laboratory unit are acceptable. For the whey powder solution, the intensity of fouling may be reduced by lowering the feed temperature to avoid conformational changes of heat-sensitive whey proteins. Lactose could be concentrated up to its solubility limit at the given temperature until lactose crystals started to form on the membrane surface. Here a higher permeate temperature could prevent crystallization due to a temperature-shift from the membrane surface to the bulk solution. In general, the application of higher feed temperatures for streams that are not temperature-sensitive could increase flux over-proportionally and achievable water recovery of such streams. The concentration reached for whole milk can probably be further increased by homogenization of milk, although limitations to pre-concentration of whole milk are given by the increasing fat content during concentration and resulting membrane wetting. Fouling did not compromise retention of organics. FTIR provided indication for organics being present in the irreversible fouling layer. Fouling behavior of other membrane materials need to be explored for treating dairy feeds and measurements to improve flux need to be proposed. The lower temperatures used for this process are readily available in waste streams and serve as a potential source of energy to drive MD. Simultaneously valuable and heat-sensitive constituents such as whey proteins can be preserved better at such temperatures.

## 5. Conclusion

MD is little studied for dairy processing, but has potential in many areas to improve product value and treat water. This work showed the performance of PTFE membranes for MD and observed the performance as a function of dry-matter concentration for decreasing complexity of the dairy stream from milk down to pure lactose. Fats appeared to create a stronger interaction leading to fouling. Whey solution showed fouling related to time, whilst skim milk solution fouling was more related to dry-matter concentration. This was a key finding for further work to design optimal operations of MD for dairy processing which will depend on the components in the stream.
